# 

**Published:** 1847-12

**Authors:** 


### PART I.—ORIGINAL COMMUNICATIONS.

ART. 1. *A Paper on the Pathology and Treatment of Fever.* By A. G. Henry, M. D., of Pekin, Ill.
ART. 2. *Fibrinous Concretions in the Ventricles of the Heart.* By G. Sprague, M. D., of Kalamazoo, Mich.
ART. 3. *Various Forms of Intermittent Diseases.* By Dr. W. H. Martin, of Rushville, Indiana.
ART. 4. *Salivation by Chemical action in the Stomach.* By J. S. Jones, M. D., of Covington, Ind.
ART. 5. *Case of Letheon.* By Asa Horr, M. D., of Dubuque, Iowa.
ART. 6. *Proceedings of the Rock River Medical Society.*
ART. 7. *Surgery in the Hospitals after the Battle of Buena Vista.* By W. B. Herrick, M. D., Prof., &c.
ART. 8. *On the use of astringent and stimulating Injections in profuse suppuration.* By D. Brainard, M. D., Prof., &c.

### PART II.—BIBLIOGRAPHICAL NOTICES.

ART. 9. *The Dental Register of the West.* Edited by James Taylor, M. D., D. D. S., of Cincinnati, O., and B. B. Brown, M. D., of St. Louis, Mo.
ART. 10. *The American Medical Almanac for* 1848.
ART. 11. *North-Western Educator.* Devoted to Education, Liter., &c.
ART. 12. *Tracts on Generation.* Number 1.
ART. 13. *A System of Surgery.* By J. M. Chelins, Doctor in Medicine and Surgery, Public Prof. of General and Opthalmic Surg., &c.
ART. 14. *Illustrations of Medical Botany.* By Jos. Carson, M.D., Prof.
ART. 15. *Report of the Trustees and Principal of the Indiana Asylum for the Deaf and Dumb;* with an exhibit of expenditure.
ART. 16. *Wood's Quarterly Retrospect of American and Foreign Practical Medicine and Surgery.*
ART. 17. *Principles of Human Physiology, with their chief applications to Pathology, Hygiene, and Forensic Medicine.* By Wm. B. Carpenter, M. D., F. R. S., Prof. &c.
ART. 18. *Lectures on the Principles and Practice of Physic.* By Thos. Watson, M. D.
ART. 19. *A New Modical Dictionary.* By D. Pereira Gardner. M. D.
ART. 20. *A Treatise on the Diseases of the Air Passages.* By Horace Green, M. D.

### PART III.—SELECTIONS.

1. On the effects of Bloodletting on the Young Subject.
2. Medical Staff of the United States Army.
3. Epidemic Cholera.
4. A case of Four Children at a Birth, complicated with hemorrhage.
5. Memorial to Congress.
6. Baptistæ Tinctoria.
7. Chloroform—a substitute fer Ether.
8. Chloroform Inhalation.
9. Result of Experiments in Boston with Chloroform.
10. Typhus or Ship Fever.

### PART IV.—EDITORIAL.

ART. 1. Rush Medical College and the National Medical Association.
ART. 2. Galvanism in Uterine Hemorrhage.
ART. 3. Quackery.
ART. 4. On the cure of Spina Bifida by Injection of the Tincture of Iodine.
ART. 5. Introductory Lectures.
ART. 6, 7, 8 & 9. Iowa Farmer's Advocate.—Union Med. Society of Northern Indiana.—Chloroform.—Obituary.

